# The HOMING method: a participatory interview tool integrating Indigenous perspectives in housing research

**DOI:** 10.3389/frma.2025.1620770

**Published:** 2025-07-31

**Authors:** James Berghan, Fiona Cram, Anna Adcock, Sarah Tawhai

**Affiliations:** ^1^Te Manawahoukura, Te Wānanga o Aotearoa, Te Awamutu, New Zealand; ^2^Katoa Ltd., Auckland, New Zealand; ^3^Centre for Women's Health Research, Te Herenga Waka – Victoria University of Wellington, Wellington, New Zealand; ^4^Community Researcher, Hawkes Bay, New Zealand

**Keywords:** Kaupapa Māori research, indigenous methodologies, participatory research, housing assessment, sense of home, housing

## Abstract

Conventional housing assessment tools often impose externally defined criteria, measuring housing quality against predetermined standards that may overlook the lived experiences and cultural values of residents. In contrast, the HOMING method is a participatory tool that centers self-determined measures of home and housing quality. Rooted in Kaupapa Māori research principles, HOMING shifts power to participants, allowing them to articulate and assess what makes a house a home based on their own lived experiences, rather than externally imposed benchmarks. The name HOMING encapsulates both “Home Of Mine”—emphasizing the deeply personal nature of home—and “housing” as an active process: what people feel, think, and do to create a home. Participants use blank wooden blocks [named *Aro Rākau* by a kuia (female elder)] to write or draw their own housing values, then collaboratively rank and discuss these through a hands-on process of building and assessing home characteristics. This method not only facilitates rich, nuanced understandings of home, but also aligns with decolonial research approaches by centering Indigenous and participant-led perspectives. This paper introduces the HOMING method, outlines its rationale within a Kaupapa Māori research paradigm, and presents case studies reflecting on its application. Through a collaborative reflective process, the paper explores how HOMING can expand housing research methodologies, making them more inclusive, reflexive, and culturally responsive.

## 1 Introduction

Principles of Kaupapa Māori research, such as aroha ki te tāngata (a love for the people), call for researchers to do our best to reduce the potential distance between “researchers” and “research participants.” Rituals of encounter and whakawhanaungatanga (process of establishing relationships and relating to others) go some way to this, yet our research tools can hold unspoken assumptions about participants' worldviews. How can research methods enable us to step with participants into their world and re-search with them, seeing through their lenses rather than imposing our own?

This paper contributes to global discourses on home and place attachment by introducing the HOMING method, a participatory research tool designed to facilitate deeper, participant-led conversations about what makes a house a home. Existing housing assessment methods often rely on externally imposed criteria, measuring housing suitability against predefined standards. In contrast, HOMING allows participants to define their own measures of home, using wooden blocks to express, rank, and discuss their housing priorities in a tactile and interactive way.

HOMING is both a concept and a process. The first three letters—Home Of Mine—highlight the deeply personal nature of home, while “-ING” emphasizes home as an active and dynamic lived experience. The method invites participants to articulate what they value in a home, rather than assuming what is important for them. Central to the method is the use of blank wooden blocks, named *Aro Rākau* by a kuia (female elder), which encourage both reflection and play.

This paper situates the HOMING method within Kaupapa Māori research, outlines its methodological rationale, and presents case studies reflecting on its use in housing research and evaluation projects. Through these case studies, we explore HOMING's potential to foster culturally responsive, participant-led housing assessment.

### 1.1 Kaupapa Māori research

The Māori world is whakapapa (genealogy), whenua (land) and whānau (familial collectives) (Cram et al., 2019), and it is through the process of whanaungatanga (kinship, relationships) that we know our world (Mead, 2003; Smith L. T., 2012). This world—te ao Māori—has been assaulted throughout the short history of the colonization of Aotearoa (New Zealand), and Western research has been a tool of colonial oppression—to relegate Māori as perpetually inferior, passive subjects (Bull, 2010; Ormiston, 2010; Rigby and Kohler, 2002; Smith L. T., 2012). An often-heard cry is that Indigenous peoples, Māori included, have been researched to death. During the 1992 Canadian Royal Commission on Aboriginal Peoples an Elder asked whether it was now time for Indigenous peoples to research ourselves back to life (Brant Castellano, 2004).

Kaupapa Māori research is a response to this call to research ourselves back to life. Kaupapa Māori literally means a Māori way. It is a response to the colonization in Aotearoa that has seen Māori (Indigenous peoples) marginalized in our own lands, as evidenced by widespread Māori-non-Māori disparities. What began in the late 1980s as Kaupapa Māori research within Māori education has spread to other disciplines (e.g., health, geography). A Kaupapa Māori inquiry paradigm sees being Māori as normal and promotes a structural analysis of Māori disparities that moves the discourse away from victim-blaming and personal deficits to more fully understanding people's lives and the systemic determinants of their health and wellness (Smith G. H., 2012).

The kaupapa or agenda of Kaupapa Māori research is making a positive difference for Māori, by privileging Māori world views, knowledge and ways of knowing, and ways of being. Kaupapa Māori research is about conducting empirical research and building theory so that it contributes to transformations that support Māori wellbeing. We are active in building the capacity of Māori to undertake research, and committed to upholding community aspirations, development, and a sovereign research agenda (Smith L. T., 2012).

In her seminal work Decolonizing Methodologies, Smith L. T. (2012) describes seven community-up research practices that frame ethical behavior in Māori and other community research settings where researchers are endeavoring to be culturally responsive (see Table 1). These principles, widely applied by Māori researchers and evaluators (e.g., Pipi et al., 2004; Cram and Phillips, 2012), are designed to reduce the distance between researchers and participants, acknowledging that traditional research methodologies have historically privileged Western imperatives of objectivity and the pursuit of “truth.” By contrast, Smith L. T. (2012) and other Māori and Indigenous researchers have challenged the idea of objectivity, choosing instead the way of many researchers working with minoritized and marginalized peoples in order make space for their voices, life worlds, concerns, and aspirations. This way includes the dismissal of the possibility of researchers being objective and instead acknowledges that researchers are loaded with biases and come to their roles as combinations of insiders and/or outsiders within our research communities. From this standpoint, they then seek to undertake rigorous and valid research.

**Table 1:** Community-up research practices (adapted from Smith L. T., 2012).

**Principle**	**Guideline**
Aroha ki te tāngata	A love for the people—allow them to define their own space and meet on their own terms
He kanohi kitea	Meet people face-to-face, and also be a face that is known to and seen within a community
Titiro, whakarongo… kōrero	Look and listen (and then maybe speak)—develop an understanding in order to find a place from which to speak
Manaaki ki te tangata	Share, host, and be generous
Kia tūpato	Be cautious—be politically astute, culturally safe, and reflective about insider/outsider status
Kaua e takahia te mana o te tangata	Do not trample on the ‘mana' or dignity of a person
Kia māhaki	Be humble—do not flaunt your knowledge; find ways of sharing it

Within this paradigm, Māori researchers begin from a position of love for the people—aroha ki te tāngata—that places research participants in the “driving seat” of the research they are being invited to be involved in Keefe et al. (1999). The role of the researcher is then to become known to the people, by being among them, looking and listening, and being generous. This does not mean the researcher is naïve. Rather they are careful and move safely so as not to trample on people's mana (status). At the same time, they remain humble and find ways to both share their own knowledge and learn from the people they engage with (Cram et al., 2018).

It is within this ethical and methodological framework that we seek out research methods that allow Māori research participants to speak on their own terms, in their own ways, without the constraints of Western epistemologies. This requires critical reflection on language, framing, and power dynamics within the research process. For example, in a study of Māori and Pacific family success, researchers first conducted open-ended interviews to allow families to define success on their own terms. These responses were then reflected back in a second round of interviews, where families were invited to select and rank key success factors that resonated with them (and this is when the family debates usually started) (Cram et al., 2020). This iterative, participant-led process ensured that the research reflected community understandings rather than imposed academic definitions.

How, then, can housing research adopt similar approaches—ensuring that Māori voices and lived experiences shape the measures of what makes a house a home? The remainder of this paper explores this question by critically examining conventional housing assessment methods, introducing the HOMING method as a Kaupapa Māori-aligned approach, and reflecting on its application through case studies.

### 1.2 Motivation for a new method to evaluate housing

Housing can be evaluated through multiple cultural and philosophical lenses, each shaping how adequacy, quality, and suitability are defined. In Aotearoa, various housing evaluation frameworks exist, including the national Housing Condition Survey and the more recent Pilot Housing Study, both of which assess the physical condition of dwellings and their financial implications for repair and maintenance (White et al., 2021; Page et al., 1995). These assessments provide critical data for policy development and housing standards, directly impacting decision-making at national and regional levels.

A growing body of research links housing quality and resident health outcomes. Studies have shown that dampness and mold contribute to respiratory illnesses (e.g., Keall et al., 2012), while crowded living conditions increase the risk of asthma and other health concerns (e.g., Antova et al., 2008). The World Health Organization (2018) has recognized housing as a key determinant of health, reinforcing the need for robust housing assessments. However, while these studies offer valuable insights into the physical and health-related aspects of housing, links to measures of more subjective and deeply personal concepts such as what makes a house a home are less clear.

A home is more than a physical dwelling—it is a space of security, stability, and control (Dupuis, 2012; Dupuis and Thorn, 1998). Ontological security, or the sense of predictability and rootedness in one's living environment, is an essential component of wellbeing and life satisfaction (Hewitt, 2010: p. 512). People who experience a strong sense of home—often associated with ownership and long-term housing stability—tend to report higher life satisfaction (Hulse and Milligan, 2014; Stats, 2020). However, perceptions of home are highly individual and influenced by cultural, social, and economic factors.

For Māori, notions of “home” are deeply relational, extending to whenua, whakapapa, and whānau (Cram, 2020). The concept of ontological security in a Māori context is inherently collective and land-based, aligning with the whakatauki (Māori proverb) “ko au te whenua, ko te whenua ko au” (I am the land, and the land is me). The word “whenua” itself means both land and placenta, reinforcing the interconnectedness of people and place. Conventional housing assessments, which focus primarily on physical infrastructure and economic viability, often fail to recognize these relational and spiritual dimensions of home.

Similar tensions between Indigenous worldviews and dominant housing systems are evident in global contexts. In North America, for example, many First Nations and Native American communities live in housing that is poorly constructed or culturally inappropriate (Patrick, 2014). In Australia, O'Brien (2011) argues that bottom-up housing approaches are needed to better reflect Indigenous community aspirations, rather than relying on top-down, state-procured models. Others support this position, emphasizing that Aboriginal self-determination is essential to addressing persistent housing inadequacies (Anthony et al., 2025; Memmott et al., 2003). Elsewhere, Indigenous peoples living away from their ancestral homelands have adapted their homes in culturally meaningful ways to support wellbeing (Faleolo, 2020). Across these diverse contexts, Indigenous peoples continue to assert values of home as relational, intergenerational, and land-based, yet these are often rendered invisible in dominant policy and evaluation frameworks. This underscores the need for Indigenous-led, culturally responsive housing research that honors diverse understandings of “home.”

Conventional housing evaluation measures in Aotearoa tend to be prescriptive, often defining success through externally imposed criteria that may not reflect the lived experiences of diverse communities. Research on Māori perceptions of home has increasingly adopted interpretive and inductive methods, allowing participants to define home in their own words (Boulton et al., 2022; Cram, 2020; Russell et al., 2023). While some housing studies have combined qualitative interviews, focus groups, and surveys (Beacon Pathway Ltd, 2010), these approaches still rely on researcher-defined measures, rather than empowering participants to construct their own assessment frameworks. Kake and Paul (2018) attempted to bridge this gap by proposing Māori design principles for housing assessment, yet their framework still prescribed fixed evaluation criteria. This raises a fundamental question: How can we move beyond prescriptive measures to methods that reduce the distance between “researcher” and “researched,” ensuing that participants retain autonomy over defining and evaluating their own housing conditions?

One way to address this challenge is through collaborative and participatory research methods, often described as “bottom-up” approaches. Participatory research emerged in the 1970s in response to the ineffectiveness of conventional, top-down research models in producing meaningful change for marginalized communities, and the term has been gaining increased attention ever since (Schubotz, 2019). These methods emphasize democratic participation, shared knowledge production, and community ownership of research processes. However, the level of participant involvement varies. For example, at lower levels of involvement, participants may only review transcripts or validate findings post-interview. Conversely, active participatory research may have participants involved in partnership with the research team, contributing to study design, data collection, and analysis.

Participatory methods are increasingly shaping housing research. For example, in 2018, Housing First used a photovoice approach, allowing participants to determine the outcomes they deemed important to be measured and discussed (Pruitt et al., 2018). Similarly, Soaita and McKee (2021) adapted photovoice for telephone interviews, enabling British tenants to articulate their tangible and intangible conceptions of home. While photovoice methodologies are powerful in amplifying participant voices, they can be time-intensive and require multiple engagement sessions (Miterko and Bruna, 2022). This presents a challenge for research projects with limited time and resources.

This paper introduces the HOMING method, a participatory approach that places power in the hands of research participants by enabling them to self-determine the parameters by which their housing is evaluated. HOMING is designed to be (1) flexible—it evolves based on participant needs and perspectives; (2) tactile and interactive—it uses wooden blocks to facilitate engagement and reflection; and (3) efficient—unlike photovoice, it is designed to be completed in a single session, making it more accessible for time-constrained participants.

While the HOMING method aligns with other community-led, participatory approaches, it offers a unique contribution by integrating both physical engagement (through blocks) and narrative storytelling to co-create definitions of home. The following sections outline the method, application, and researcher reflections on using HOMING in housing evaluation.

## 2 HOMING method: protocol for implementation

**Figure 1:**
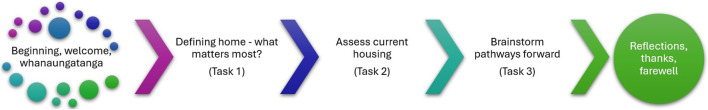
Flowchart summary of steps when using the HOMING method process with participants.

The HOMING method is a hands-on, interactive participatory tool designed to facilitate conversations about what makes a house a home. Participants use wooden blocks to define their own housing criteria, engage in ranking exercises, and reflect on their current housing realities through a traffic-light assessment. A summary of the process is shown in Figure 1.

### 2.1 Participants

Anyone can be a participant in this method, including people as individuals or as groups (e.g., whānau). Groups can be made up of people of similar ages or people from different generations. If people are participating in groups, then there is an opportunity for them to come up with collective ideas about what makes a dwelling a home. This can take time.

### 2.2 Materials needed

Users of the method need: (a) 10 blank wooden blocks per participant or group of participants (for writing or drawing housing values); (b) pre-labeled example blocks (optional, to provide prompts); and (c) three painted blocks—red, yellow, and green (used for traffic-light assessment).

### 2.3 Beginning

When used, this method should have an appropriate beginning. Participants are welcomed to the project and given an opportunity to introduce themselves to other participants. The researcher should check in with the group of participants to make sure it is okay for them to take pictures and recordings of the session.

The importance of this phase cannot be understated: the beginning is about providing a safe space for participants to feel like they can openly express their opinions throughout the exercise without prejudice or judgement, and that like other participatory methods, the HOMING method allows for conflicts and differences of opinion (Bergold and Thomas, 2012).

#### 2.3.1 Task 1: defining home—what matters most?

**Figure 2:**
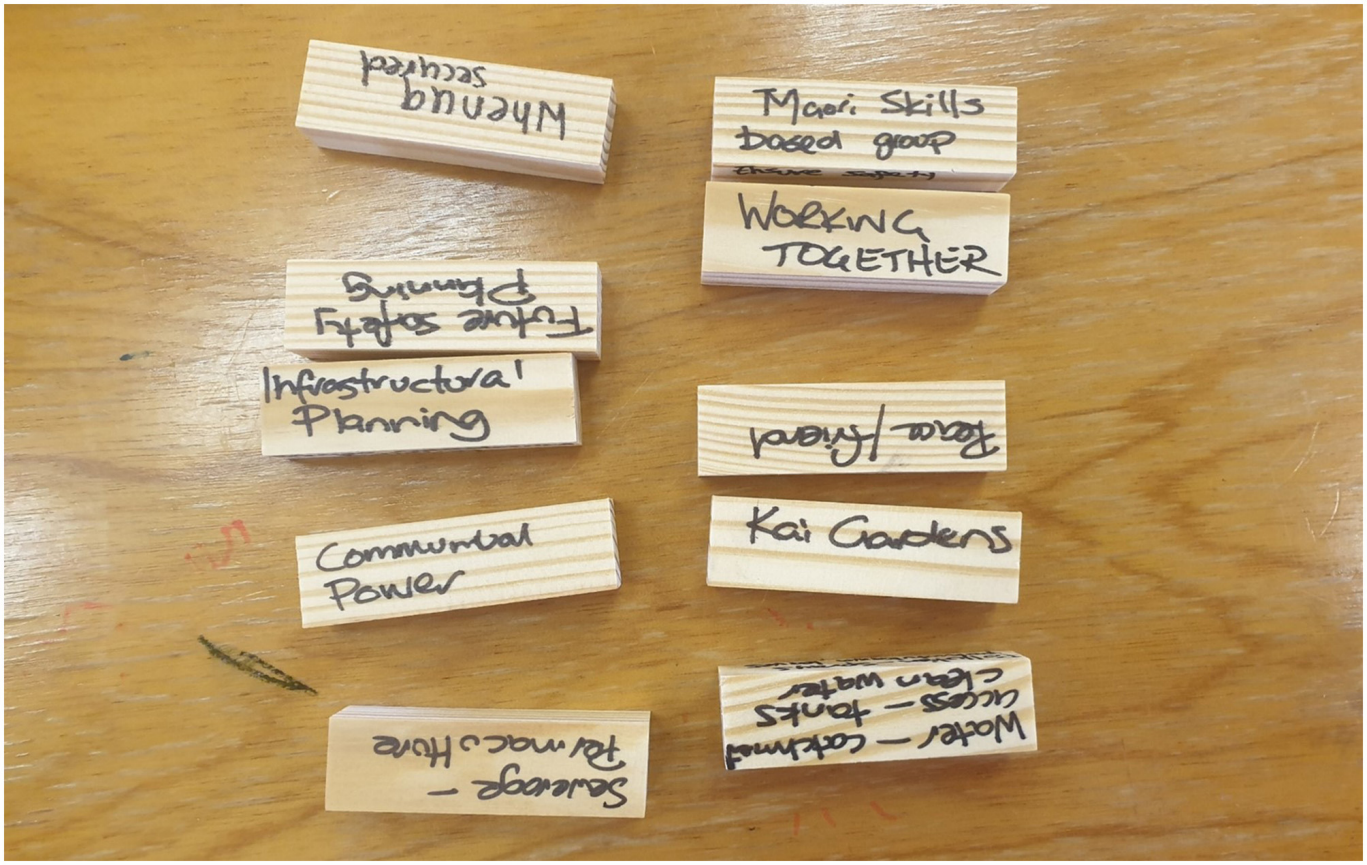
Example of wooden blocks with ideas written on them.

The first task is to come up with 10 things that are important for making a dwelling a home. Each of the 10 characteristics is then written on a wooden block (see Figure 2 for an example).

Next a vertical block tower is built, with one block being placed on another with the writing facing front. The blocks are sorted so that the least important characteristic is placed at the base of the tower, up to the most important characteristic being the last block placed on the top of the tower. This will give groups an opportunity for more discussion and individuals the chance to deliberate the importance of the things they have written down. The tower, when built, illustrates the rank participants give to their characteristics.

The researcher(s) should circulate during group activity to take pictures (if permission obtained) and record their observations about interactions and discussions. If there are individual participants researchers might engage them in conversation about what they are contemplating when doing this task. It may also be possible for researchers to “drop in” to group conversations and ask questions about how they are getting on. It is important that pictures of the final towers are taken. Table 2 contains some suggested instructions for participants.

**Table 2:** Suggested instructions for participants (task 1).

**Task 1.1 Deciding what makes a home**
**“Having a home can mean different things to different people. What we'd like you to do is decide what for you (as an individual/as a group) are the ten most important things that make a dwelling a home**.
**Each person/group has been given ten blank blocks, along with 2 or 3 blocks that have something already written on them. These additional blocks are to show you what we mean by writing on the blocks, and you can use them as part of your ten blocks if you find they belong there. If they don't, then don't use them. It's up to you**.
**You'll have around 20–30 minutes to decide what the ten most important things are for you, that make a dwelling a home. See if you can capture each thing as a keyword or short idea that will fit on a block—no essays please! [Note: individual participants may need less time]**
**For groups: please don't write anything on the blocks until you've come up with a list of 10 agreed upon things. Then you can write these ten things on the blocks.”**
**Task 1.2 Building a tower**
“Now that you've decided on your 10 most important things about what makes a home, I want you to build a vertical tower; that is, a tower that's ten blocks tall with one block placed on top of another.
To build this tower, I want you to order the 10 blocks you've got—from the least important thing on the bottom of the tower to the most important thing on the top of the tower.
You'll have around 20 minutes to decide on the order and to build your tower.”
**Task 1.3 Feeding back**
“We're going to go around the room now and hear back from everyone about their ten most important things and their order of importance. Tell us a little about each block, for example, how you've defined what you've written if it's not obvious from what you've been able to fit on a block.
For groups: I'd especially like you to tell us about any particular points of agreement you had as a group, and any particular points of disagreement.”

#### 2.3.2 Task 2: assess current housing

The second task is for participants to individually or in household groups to rate the performance of their current dwelling using their list of the 10 most important characteristics. This rating is done by participants building another tower on a traffic light base. When they get the painted blocks, they put them down first in a traffic light row—red, orange, green. They then use their 10 blocks to build a second tower on top of this base:

- Building on the red base indicates that these things are absent from their present dwelling;
- Building on the orange base indicates that these things are partially present; and
- Building on the green base indicates that these things are fully present.

As with Task 1, the researcher(s) should circulate during group activity to take pictures (if permission obtained) and record their observations about interactions and discussions. If there are individual participant researchers might engage them in conversation about what they are contemplating when doing this task. It may also be possible for researchers to “drop in” to group conversations and ask questions about how they are getting on. It is important that pictures of the final towers are taken. Some suggested instructions for task 2 are given in Table 3.

**Table 3:** Suggested instructions for participants (task 2).

**Task 2.1 Traffic light base**
**I'm now going to give you each a set of three painted blocks and I want you to put these down in a row in front of you so they're like a traffic light—red, orange, green**.
**Task 2.2 Current dwelling assessment**
Now you're going to build a second tower, using the same 10 blocks as before. This time I want you to build a tower that's three blocks wide with the bottom row of the tower being the traffic light blocks.
For each of your own 10 blocks I want to you decide whether or not that thing that you've written on a block about what makes a home is present in your current dwelling. So if the first block I pick up is, say, warmth (heating) I'll decide whether I put this block on top of the red block—because my current place is not warm, or on the orange block—because my current place is warm sometimes, or on the green block—because where I currently live is warm all the time.
Any questions/pātai?
I'll give you 10 minutes to do this, and we'll check in to see if this is enough time.
**Task 2.3 Feeding back**
Let's go around the room again and hear back from each of you about 1–2 of the things that are absent from your current dwelling and (hopefully) 1–2 of the things that are there all the time.

#### 2.3.3 Task 3: brainstorm pathways forward

Task three is about identifying the challenges to and solutions for people's lack of access to the things that make a dwelling a home. This task is done as a big group. Participants' responses can be noted on a whiteboard or PowerPoint slide in a three-column table with headings: “Home,” “Challenges,” “Solutions.” Some suggested instructions for participants are provided in Table 4.

**Table 4:** Suggested instructions for participants (task 3).

**Task 3.1 Challenges and solutions**
We're doing to stay in a big group for this last discussion. I want you to think about the things you've come up with about what makes a home, and name some of the local challenges for people having this where they live. So, if I go back to “warmth (heating)” as an example, a challenge might be people's inability to afford the cost of electricity. Then when we know the challenges, we're going to brainstorm solutions.
• So, who'd like to start with one of their blocks?
• What are some of the challenges to people having this?
• Do you have ideas about solutions?

Proceed like this until time is up, working through characteristics from their blocks, local challenges and ideas for solutions. This exercise leaves people feeling like there are solutions, and that it is important for people to have somewhere that is a home for them.

### 2.4 Finishing up

The final task can be a round of checking in with people about how they have found the exercises, followed by appropriate thanks and farewells.

### 2.5 Analysis

The data gathered from this method includes:

- Researcher notes taken during tasks 1 and 2. Group discussion notes taken during task 3, and any notes taken about group feedback when the session closes. These notes can be either taken discreetly and/or written up immediately after the session while still fresh in the researchers' minds. A debriefing of researchers following the session is another source of notes, as researchers debrief and compare what they've seen, heard and noted.
- Photographs of the towers built in tasks 1 and 2, linked to researcher notes of group/individual feedback about tasks and outcomes to the larger group.
- Any recordings (audio, visual) taken (with participant permission) during the session. It may be possible, for example, to audio record groups decision-making about their top 10 characteristics of a home.

There are at least two components in the analysis. The first is what participants think makes a dwelling a home. A theming of the blocks collected following a session will allow for a count of the most frequently chosen characteristics as well as some of the more unique characteristics. Alongside researcher notes, this count will be supplemented by group and individual deliberations when deciding on these characteristics.

The ranking or priority given to these characteristics can be gained from pictures of the towers built in task 1. This will allow some weighting to be given to different characteristics to compare frequency and priority. For example, it may be that the most frequently mentioned items are less prioritized in the tower builds.

The pictures of task 2 towers provide assessment data about whether or not participants have the things they value most about what makes a home. Task 3 notes supplement the intel about why the achievement of characteristics might be challenging. While not necessarily related to their own circumstances (unless identified as such by them), task 3 provides insights while not putting participants on the spot. The solutions suggested in task 3 can be themed into recommendations for improving local housing.

## 3 Methodological reflexivity

The HOMING method originated through critical reflection on gaps observed in existing housing evaluation tools, particularly their inability to capture subjective and culturally-nuanced understandings of home. Early iterations of the method were piloted informally within smaller wānanga settings (akin to a focus group setting), where participants trialed writing and stacking blocks. These preliminary sessions affirmed the potential for tactile, open-ended engagement to prompt deeper reflections on housing and belonging. However, they also revealed important dynamics around facilitation—for instance, that some participants sought to “please” researchers by arranging blocks in neat towers, while others naturally subverted expectations through creative arrangements. Recognizing these dynamics prompted adjustments in facilitator instructions to explicitly affirm participant autonomy and creativity. Throughout the development of HOMING, an emphasis was placed on minimizing researcher-led framing, allowing participants' own worldviews, priorities, and modes of expression to lead the process.

The HOMING method has been trialed by the authors and other collaborators across a range of housing research projects within the Affordable Housing for Generations (AHfG) research program. AHfG is a multi-year program funded by the Building Better Homes, Towns and Cities National Science Challenge, and is co-led by Dr. Fiona Cram and Dr. Kay Saville-Smith. This section of the article draws from our collective critical reflections of trialing the method to date with over 100 participants across Aotearoa, in a range of research settings, between 2021 and 2024.

### 3.1 Lesson 1: people are imaginative

While our original intent was for participants to build a single-file tower (with 10 blocks stacked directly on top of one another), very rarely was this the type of tower built by participants. Some towers were two or four blocks wide, others were built in a pyramid shape. Others were circular. This was a reminder that there needs to be a balance between providing (restrictive) direction and allowing for creativity and flexibility. Asking participants why they had arranged their towers in the ways they had often opened the door for further discussion and challenged our initial idea of rankings being singular and ordered (i.e., thinking that two things could not be ranked equally).

#### 3.1.1 Vignette: an insight into the spatial arrangement of block towers

**Figure 3:**
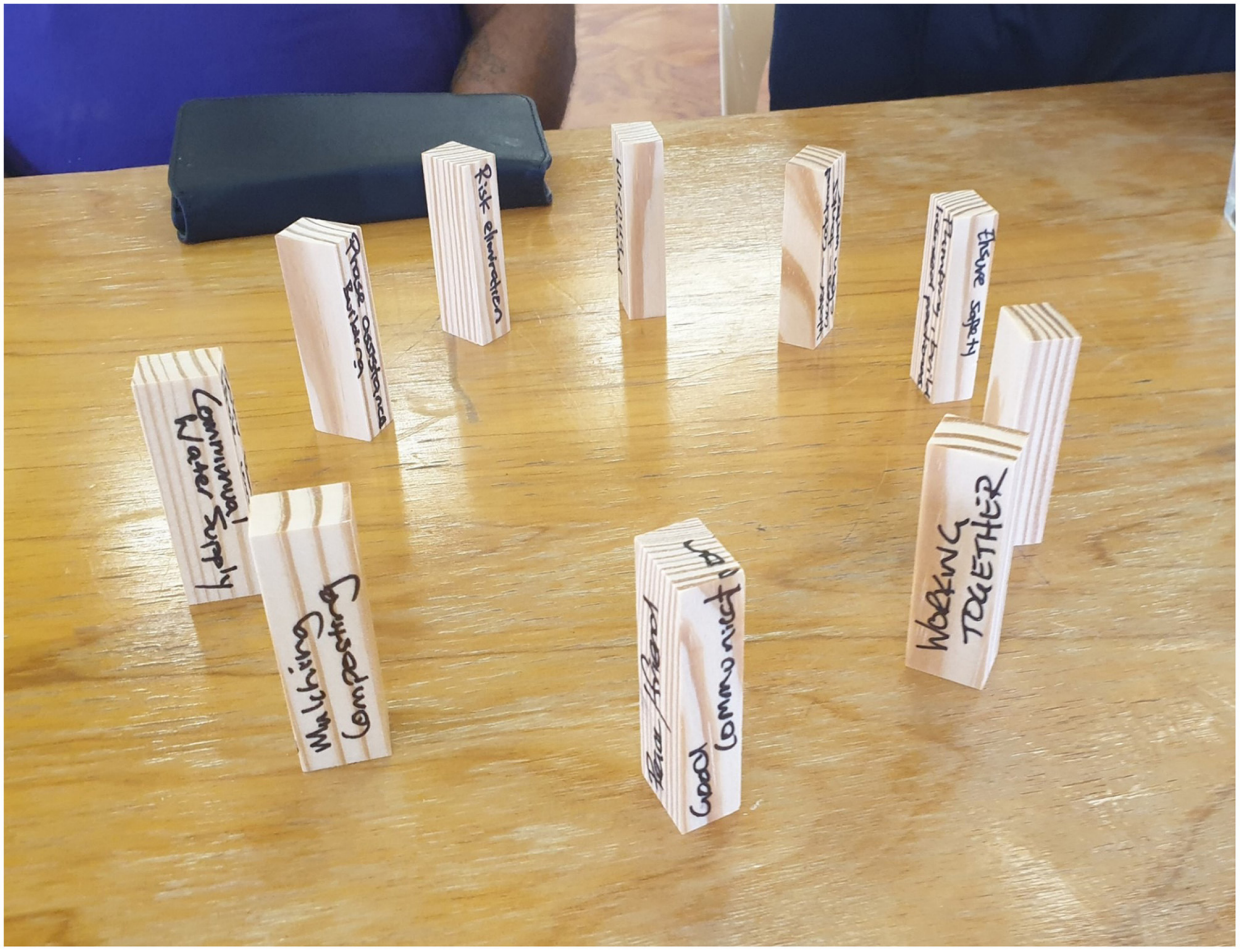
These blocks were arranged in a circle to represent “stay lines” supporting the kāinga.

At a research wānanga in the Bay of Plenty, a group of participants placed their blocks on their ends and arranged them in a circle (see Figure 3). When queried by the researchers about this arrangement, the group likened their blocks to “stay lines.” Just as a stay line (or “guy wire”) can be used to support a power pole or a yacht's mast, the values inscribed on this group's blocks were considered to all be equally important in supporting and holding up the kāinga.

### 3.2 Lesson 2: it takes time

Some individuals and groups can complete the block naming and the stacking quite rapidly, whereas for others, it can take a lot longer to devise lists and to negotiate their rankings (especially so for groups). Again, this was a reminder that this method demands flexibility. It might take up all the time you have scheduled just to construct the list, for instance.

What's more, asking a question such as “what makes a house a home?” can be challenging to think about for some people, especially when put on the spot. Where possible, participants can benefit from knowing the question that will be asked ahead of time, to have some time to consider different ideas and values. Even so, participants whose current housing is precarious and/or of poor quality may find it difficult to contemplate the lack of expression of the things they value most in their current home. Participants should therefore always have the option of opting out of participating in any of the tasks described above.

### 3.3 Lesson 3: some participants will need different levels of guidance

Depending on the context and the background of the participants, participants might need different levels of guidance to carry out the activity. For instance, students in an urban design class who have been studying housing during the semester were relatively well-grounded in the context of housing and needed little guidance and few prompts. In other contexts where this exercise is the first instance of housing being discussed in a research context amongst participants, they might need more guidance. Having a few pre-named blocks can be helpful here, that participants can choose to add to their set if they want. Likewise, sometimes participants may find choosing 10 important things challenging and will want to settle for a smaller number that they feel confident about. For instance, in one case study, two participants on two different occasions opted to choose eight important things.

Similarly, there are differences between running the exercise with individuals or with groups. Working in pairs or groups allows for discussion and negotiation. With individuals, it can be helpful if the “researcher” can chat to participants along the way to provide prompts or guidance if they may be feeling stuck.

In either case, the importance of taking time for introductions and scene-setting cannot be understated: making sure everyone knows the purpose of the study and the researchers being able to ask different questions or prompts to get participants to think of words to write on their blocks.

### 3.4 Lesson 4: follow the lead of participants—you can (and should) be adaptable

**Figure 4:**
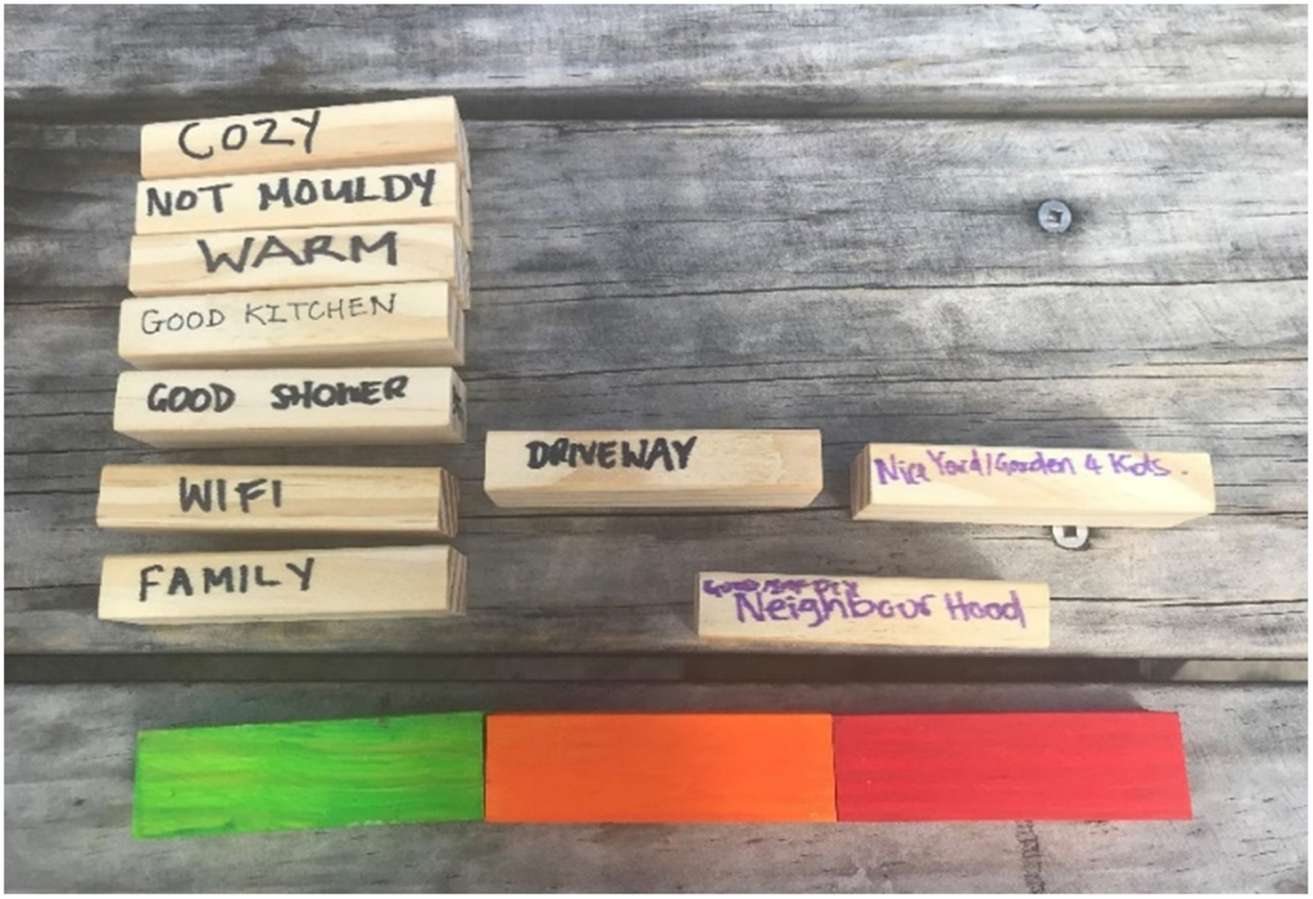
Blocks arranged outside on a park bench—where the interview took place.

The need to be flexible must be emphasized. Participants will all come to the activity with different bandwidth. To ensure comfort and confidence, it is imperative to follow the lead of the participants. Find a space and time that is suitable, be it in a home, in an office, or even outside (weather permitting—see Figure 4). Some participants may decide that they prefer to contribute to the activity online through platforms such as Zoom (for instance, due to COVID-19 precautions), which requires either sending the materials ahead of time or creating a system of collecting the information online. Depending on who is being interviewed, there may be other people present, such as young children or support people. Being able to pivot to meet the needs of participants shows that their contributions are valued, and they come to the activity on their own terms.

#### 3.4.1 Vignette: being inclusive of whānau

**Figure 5:**
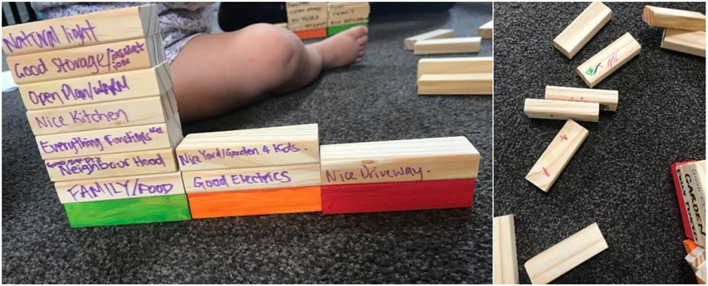
Block activity with pēpi—before and after.

At an interview with two young māmā (mothers) in Horowhenua, we sat on the floor with one of the māmā's pēpi (an infant and a toddler) in their living room (see Figure 5). The pēpi wanted to take part too, drawing on several blocks happily. The towers did not stay up for very long either! Being inclusive of the pēpi meant the māmā could engage in the activity without concern about what their pēpi were doing.

#### 3.4.2 Vignette: making the activity work online for whānau

**Figure 6:**
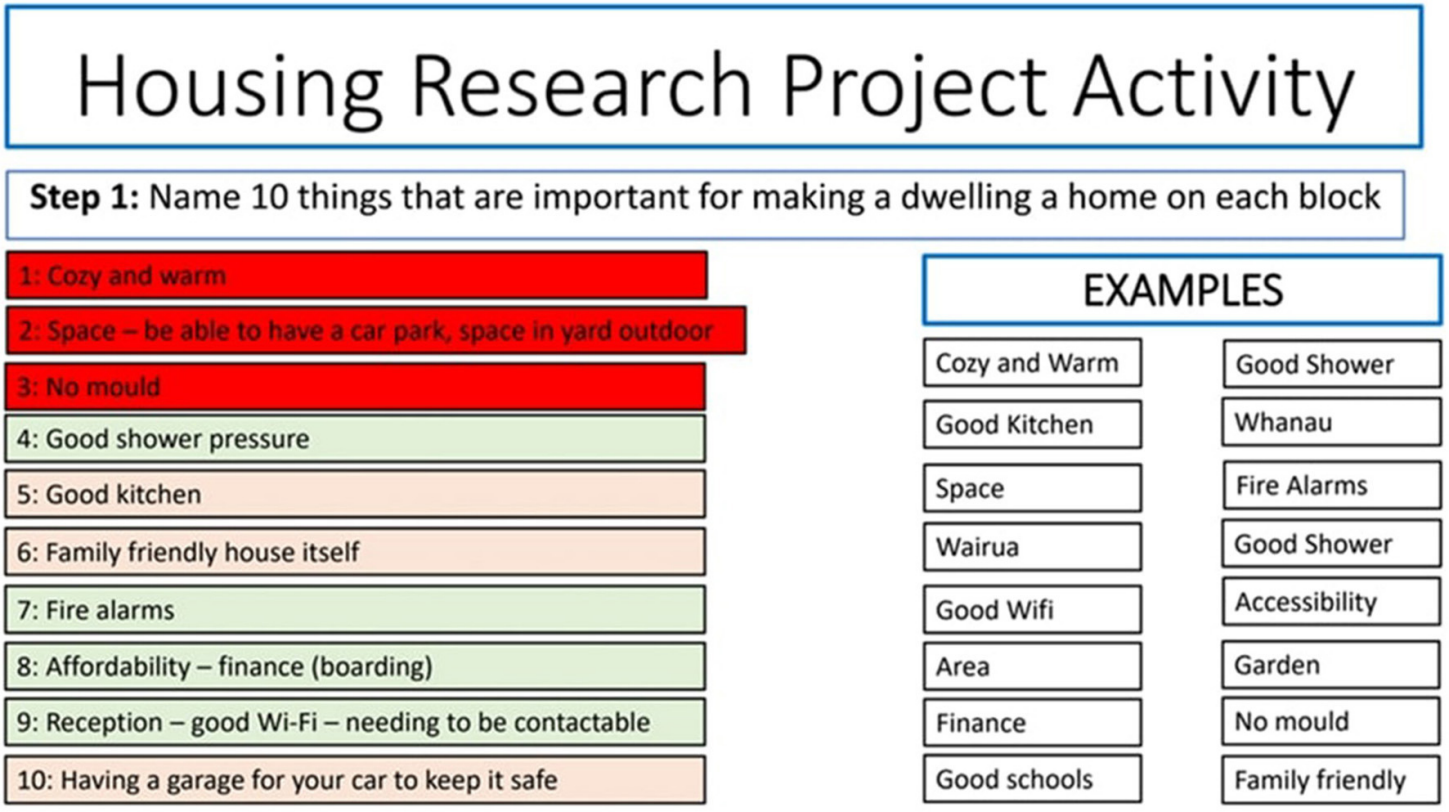
PowerPoint slide used for online participants showing “virtual” blocks numbered and shaded.

During a study with young māmā in Wellington, most māmā chose to take part through online interviews. A PowerPoint slide was set up with examples and boxes for each participant's “important things” to be shared and recorded on. Then the boxes were numbered, and finally, colored in according to their traffic light color (see Figure 6).

## 4 Discussion

The HOMING method was developed to provide a tangible and interactive means for participants to express their feelings about home. The decision to use blocks was based on their versatility and the ease with which they can be manipulated to represent various aspects of home. Initial pilot studies indicated that participants found the blocks engaging and that they facilitated rich, detailed narratives.

The use of blocks appeared to democratize the interview process, allowing participants to take an active role in the conversation. However, this also introduced a dynamic where some participants felt pressure to create “correct” or “impressive” arrangements. This was mitigated by emphasizing that there were no right or wrong ways to use the blocks.

### 4.1 Blocks as a metaphor for whakapapa

While wooden blocks provide a tool for play, building towers from blocks also acts as an analogic play on the Māori concept of whakapapa. The term whakapapa is commonly defined in its noun form, to mean genealogy. However, in verb form, whakapapa (tia) can mean “to place in layers, lay one upon another” (Te Aka Māori Dictionary, 2023). Building a tower of wooden blocks then becomes a physical representation of a participant's “whakapapa of home”: the layers that make a house a home for that person or group.

### 4.2 Blocks as a medium for communication

The kinesthetic element of the HOMING method allows participants to articulate their thoughts, experiences and perspectives on what makes a house a home in different ways. While there is still some reliance on literacy (to label blocks—though participants are encouraged to write or draw on their blocks in any way or in any language that makes sense to them), the limited space on the blocks forces participants to coalesce ideas down to one or two overarching ideas or principles to fit on the block. While participants still share their reflections verbally and in a similar way to an interview or group interview-style setting, the blocks can act as a mediator for participants to engage in, and to communicate and negotiate their ideas before sharing. Similar principles underpin photovoice as a visual research method which offers photos as an alternative medium for communication (e.g., Vaughan, 2016). Unlike photovoice, which requires extended engagement and technical skills, HOMING provides a simple, interactive tool that can be used in a single session while still capturing deep insights.

### 4.3 Blocks as a tool for reflection and play

Using reflection and play, the method elicits deep understandings of home, particularly through the negotiation that can happen between participants when ordering and scoring blocks. Research directly comparing participant responses and ratings from the HOMING method to more conventional housing assessment measures could helpfully validate if the depth and breadth of responses are in fact different between the two.

### 4.4 But is it a Kaupapa Māori method?

A critical question is whether the HOMING method aligns with Kaupapa Māori research principles. Paipa et al. (2015) describe five principles they use to identify, screen and, if needed, revise evaluation methods so they are culturally responsive for Māori (Table 5). We reflect on each of the five principles below, to argue that the HOMING method does indeed align with Kaupapa Māori principles.

**Table 5:** Principles of a Kaupapa Māori research method (from Paipa et al., 2015).

**Principle**	**Guideline**
Whakapapa (genealogy)	Participants can make connections, including with the past and future
Whakawhanaungatanga (to make or strengthen relationships)	The method includes the ability to capture the fullness of relationships, such as people's care and support for one another
Whakawātea (clearing the way)	The method's ability to respond to the unique ways people will engage, including language modes
Whakaae (acceptance)	Reaching agreement about the sharing of information, ownership of knowledge shared, and participants' consent to be involved in the research
Whakamana (the enhancement of mana)	Whether participants gain something from their involvement in the research

#### 4.4.1 Whakapapa (genealogy)

The HOMING method facilitates whakapapa by encouraging connections across time, places, and relationships. The method appropriately begins with time for introductions, both to the research and to one another. The importance of starting with this process of establishing connections and relationships cannot be understated. Tiakiwai (2015) describes how connection building allows participants to go beyond a researcher/researched construct to form a much more personal and deeper relationship that extends beyond the research activity. The sharing of whakapapa is encouraged by all in the room, to be able to make connections to people and places; past, present, and future.

#### 4.4.2 Whakawhanaungatanga (to make or strengthen relationships)

The premise of the HOMING method is that it places the naming of evaluation measures in the hands of the participants. In doing so, it opens up space for connections to be expressed. Universal methods of housing evaluation have the risk of unintentionally excluding certain measures, depending on their own normative assumptions about housing and home life. For instance, a study of household crowding with Eabametoong First Nation found that residents' self-assessed experiences of crowding brought forth knowledge which was not visible in existing frameworks based on an assumed universal norm (McCartney et al., 2021). In a similar vein, the openness provided through blank blocks makes room for new and unexpected words or principles to emerge.

When used in group settings, the HOMING method demands discussion and negotiation amongst participants to co-create their list of principles. Through this negotiation and collective meaning-making, participants can strengthen connections with one another as they learn what is important to others, uncover shared values and principles, or explore areas of difference.

The act of building towers also allows for relationships and connections to be made in the research and amongst participants. When participants are asked to build a tower from their blocks, they would arrange them in ways that made sense to them. Often, the form and shape of towers were expressions of connections and relationships between the different principles which had been put forward. Those relationships may not have been as clearly expressed if explored through more conventional methods such as an interview or focus group.

#### 4.4.3 Whakawātea (clearing the way)

The HOMING method goes some way toward supporting whakawātea. HOMING recognizes the context dependent, situated knowledge that participants bring with them. As noted above, the blocks can act as a tool for reflection and play, as well as a medium for communication. Incorporating this practical element helps the activity to feel less like a tool of inquiry. Participants can write words or phrases on the blocks, and in whatever language makes the most sense to them, but in many cases, the discussion that participants share to explain their blocks is the most salient.

#### 4.4.4. Whakaae (acceptance)

As outlined in the protocol, in setting up the HOMING method, the researcher/s and participants should discuss what information will be collected as part of the process (including any information participants would prefer not to be shared), what will happen with that information, and gain informed consent to proceed with the method. Each stage of the HOMING method also involves a “feeding back” stage, where participants can choose how much they would like to share in narrative with others.

Kawharu et al. (2023) call for improved institutional processes, particularly around ethics, which could (and should) more appropriately recognize and compensate Māori communities for their role in research and co-design.

#### 4.4.5 Whakamana (the enhancement of mana)

The HOMING method recognizes that participants are in the best position to determine the housing outcomes that are important to measure. In doing so, the method aims to empower participants by showing them that their thoughts and knowledge are important and valuable.

In these ways, HOMING provides a decolonial research tool that challenges Western housing evaluation frameworks by centering Māori and participant-led assessments.

### 4.5 Further and future research

This article describes the design of a research method, which, from researcher reflections, suggests that it could have benefits for participants. As Foster-Fishman and others suggest, though, further research is needed to empirically test the impact of this method:

An explicit analysis of the impact of our research methodologies will help to move this discussion beyond the question of “Does our research have an impact?” to “In what ways are individuals and communities impacted by our research?” (Foster-Fishman et al., 2005: 276).

While our reflections implicitly draw from participant feedback on the method, incorporating participant feedback more explicitly would valuably supplement researcher reflections and inform future revisions of the method.

To date, we have trialed the HOMING method in the context of evaluating houses. The method could, though, be trialed as an evaluation tool outside of a housing context; opening the scope for specific research groups or participants to determine what things or processes they would like to collectively evaluate. Such an extension is especially relevant in Indigenous research contexts, where relationships with the built environment are deeply embedded in cultural identity, values, and collective wellbeing. For Māori, spatial contexts such as kāinga (home, village) and papakāinga (collective Māori housing, typically on ancestral Māori land) are not only physical forms but also relational and spiritual constructs, shaped by tikanga (customs) and whakapapa (Awatere et al., 2008). Similar Indigenous worldviews globally emphasize place-responsive, kin-centric relationships with built and natural environments such as Native American approaches to establishing contemporary villages by reoccupying ancestral homelands (Glenn, 2021) or Aboriginal Australian design principles that center kinship, storytelling, and connection to Country (Kombumerri and Hromek, 2021). As such, evaluating spaces like neighborhoods or community infrastructure through pre-defined, Western metrics often overlooks Indigenous spatial values. Extending the HOMING method into these contexts could allow participants to articulate and evaluate space on their own terms, potentially contributing to more culturally resonant understandings of urban space and spatial justice.

Given its emphasis on participant-led definitions and values, the HOMING method may also be of interest to other Indigenous researchers (e.g., Enari et al., 2024), or to communities seeking self-determined approaches to evaluating space, wellbeing, or place-based outcomes. Its flexibility and tactile, narrative-driven format make it adaptable to diverse cultural and political contexts, particularly where dominant evaluation frameworks fail to capture relational or collective understandings of place. For instance, as Refiti (2014) explores in the context of Samoan cosmogony and built form, Indigenous spatial concepts such as vā, mavae and tofiga articulate deeply relational understandings of space, identity, and social order that diverge from Western framings of architecture and home. In such contexts, a participant-led evaluation tool like HOMING could enable more culturally grounded engagement with the built environment.

## 5 Conclusion

In this article, we posited that dominant methods of housing evaluation, at least in Aotearoa New Zealand, tend to predetermine the indicators of success. These tools or methods perpetuate normative assumptions of what a “good” home should look like by prescribing the measures that the house is assessed against. While this is suitable for some objective measures of housing quality, we argue that subjective assessments (such as “what makes a house a home?”) are better determined through methods that allow for that subjectivity to be expressed by research participants, subjectively.

The insights gained from this study have significant implications for researchers, practitioners, and policymakers globally. For instance, the HOMING method can be adapted to explore housing preferences in diverse cultural contexts, enabling researchers to co-create housing assessments that reflect Indigenous, migrant, refugee, or marginalized community perspectives rather than relying solely on Western-centric models. Similarly, while this study is grounded in the unique context of Aotearoa New Zealand, the concept of “home” and the processes through which individuals articulate their sense of belonging and identity within their domestic spaces are universally relevant. The HOMING method's flexibility and participant-led structure offer opportunities for adaptation across diverse cultural, social, and national contexts, supporting more inclusive and culturally-responsive housing research worldwide.

This paper introduces and explains the HOMING method, a participant-oriented method of housing assessment and evaluation which seeks to shift the power from the researcher and place it in the hands of those living in the homes being evaluated. The method centers the residents themselves and seeks to support them to exert their autonomy as research participants to construct and impose their own success measures, rather than someone else's. Importantly, we are not suggesting this method replaces existing housing assessment tools. Rather, we posit that it can be used to supplement existing methods in ways that may yield knowledge more appropriate to the context. Future research should empirically test the HOMING method's effectiveness by comparing its insights with those generated by conventional housing assessment tools. Further, applications could explore its adaptability beyond housing, such as evaluating community wellbeing, urban design, or neighborhood relationships. By centering participant agency and self-determined housing values, HOMING has the potential to transform housing research, creating more inclusive, reflexive and culturally responsive approaches.

## Data Availability

The original contributions presented in the study are included in the article/supplementary material, further inquiries can be directed to the corresponding author.

## Glossary

**Aro rākau**: literally, wooden blocks; a name given to the blocks used in the HOMING method.

**Aroha ki te tāngata**: love for the people, a principle of Kaupapa Māori research.

**Kāinga**: home, address, village, settlement.

**Kaupapa**: topic, policy, matter for discussion.

**Kaupapa Māori**: Māori approach, incorporating the knowledge, skills, attitudes, and values of Māori society.

**Kuia**: elderly woman, grandmother, female elder.

**Māmā**: mother, mum.

**Mana**: prestige, authority, control, power, influence, status, mana is a supernatural force in a person, place, or object.

**Māori**: normal, usual, common, ordinary, indigenous New Zealander, a new word resulting from Pākehā contact in order to distinguish between people of Māori descent and the colonizers.

**Pākehā**: New Zealander of European descent, introduced from or originating in a foreign country.

**Papakāinga**: housing complex, typically located on ancestral Māori land, and can include other activities associated with the nature and function of the papakāinga such as a marae, shared gardens, or commercial activities.

**Pēpi**: baby, infant.

**Te Ao Māori**: the Māori world.

**Tikanga**: the customary system of values and practices that have developed over time and are deeply embedded in the social context.

**Wānanga**: (as a verb) to meet and discuss, deliberate, consider; (as a noun) seminar, conference, forum.

**Whakaae**: to agree, consent, accept, approve.

**Whakamana**: to give authority to, to give effect to, validate, the enhancement of mana.

**Whakapapa**: (as a verb) to place in layers, lay one upon another; (as a noun) genealogy, genealogical table, lineage, descent.

**Whakataukī**: proverb, significant saying, aphorism.

**Whakawātea**: to clear, free, dislodge, purge, get rid of.

**Whakawhanaungatanga**: process of establishing relationships, relating well to others.

**Whānau**: extended family, family group, the primary economic unit of traditional Māori society.

**Whanaungatanga**: relationship, kinship, sense of family connection, a relationship through shared experiences and working together which provides people with a sense of belonging.

**Whenua**: land, ground, territory, domain (also means placenta, afterbirth).
